# Childhood-onset bullous systemic lupus erythematosus

**DOI:** 10.1186/1546-0096-12-S1-P335

**Published:** 2014-09-17

**Authors:** Daniela Lourenço, Roberta Gomes, Nadia E Aikawa, Lucia Campos, Ricardo Romiti, Clovis Silva

**Affiliations:** 1 Pediatric Rheumatology, Faculdade De Medicina Da Universidade De Sao Paulo, Sao Paulo, Brazil; 2 Dermatology, Faculdade De Medicina Da Universidade De Sao Paulo, Sao Paulo, Brazil

## Introduction

Bullous systemic lupus erythematosus (BSLE) has rarely been described in pediatric lupus population and to our knowledge the prevalence of childhood BSLE has not been reported.

## Objectives

To evaluate the prevalence and describe cases of BSLE in childhood-onset SLE (c-SLE) patients.

## Methods

From 1983 to 2013, 303 c-SLE patients were followed at the Pediatric Rheumatology Unit of the Children´s Institute of Hospital das Clínicas da Faculdade de Medicina da Universidade de Sao Paulo. Three of them (1%) presented BSLE and are described herein.

## Results

### Case 1

A 10-year old boy presented fever, arthritis and generalized tense vesicles and bullae on the face, oral mucosa and trunk. Biopsy of a vesicle showed a subepidermal blister with neutrophilic microabcesses in dermal papillae and perivascular lymphocytes and neutrophils infiltration. Direct immunofluorescence (DIF) revealed IgG/IgA/IgM deposits at BMZ. After 1 month, he presented oral ulcers and pleural effusion. Laboratory findings showed hemoglobin 9.3g/dL, WBC 7,300/mm³(73%neutrophils, 21%lymphocytes), platelets 398,000/mm³, C3 45mg/dL and C4 6mg/dL. Urinalysis showed leukocytes 1,000/ml and erythrocytes 21,000/mL. Immunological tests showed positive ANA 1:640, anti-dsDNA, anti-Sm, anti-Ro/SSA and anti-La/SSB autoantibodies. He fulfilled Camisa and Sharma diagnostic criteria. At that moment the SLEDAI-2K score was 18. He was treated with methylprednisolone pulse therapy (30mg/kg/day for 3 days), followed by prednisone (1.0mg/kg/day), hydroxychloroquine and dapsone. After one month, the vesicles and bullous lesions improved immensely.

### Case 2

A 10-year-old girl presented recurrent bullae and vesicular lesions on the trunk for two months. Two months later, she presented oral ulcers, arthritis, pericarditis, and arterial hypertension. A skin biopsy showed neutrophilic bullous dermatitis with subepidermal cleavage. DIF revealed deposits of IgG/IgM/IgA/C3 at the BMZ. Hemoglobin was 10.9g/dL, WBC 7,590/mm³ (87%neutrophils, 9%lymphocytes) and platelets 354,000/mm³, C3 38mg/dL and C4 3mg/dL. Proteinuria was 0.67g/day. Immunological tests showed positive ANA 1:1280 and positive anti-ds DNA, anti-Sm, anti-Ro/SSA autoantibodies. The renal biopsy showed focal proliferative lupus nephritis. The SLEDAI-2K score was 24. She was treated with methylprednisolone pulse therapy, followed by prednisone, monthly intravenous cyclophosphamide (500-1000mg/m^2^) and hydroxychloroquine. After 4 months, she progressively improved, with complete resolution of bullous lesions with residual pigmentary changes.

### Case 3

A 6 years girl, presented with fever and vesicles and bullous lesions on the neck, lower limbs, and oral mucosa for 2 months. Skin biopsy showed subepidermal blister with neutrophil inflammation in the upper dermis and DIF showed deposition of IgA, IgM and IgG. Two months later, the patient developed eyelid edema and oral ulcers. Hemoglobin was 7.9g/dL, ANA 1:200 and positive anti-Sm and proteinuria was 0.6g/day. Renal biopsy revealed mesangial proliferative lupus nephritis. She received methylprednisolone pulses, prednisone, hydroxychloroquine and dapsone. After 4 months, she presented progressive improvement of the bullous lesions.

## Conclusion

In the last 30 years, the prevalence of BSLE in childhood was 1% in our tertiary University Hospital. Vesiculobullous skin involvement was the first disease manifestation in all three cases. A diagnosis of SLE should always be considered in children with recurrent tense vesiculobullous lesions especially with systemic manifestations and specific autoantibodies.

## Disclosure of interest

None declared

